# Muscle Protein Synthesis with a Hybrid Dairy and Plant-Based Protein Blend (P4) Is Equal to Whey Protein in a Murine Ageing Model after Fasting

**DOI:** 10.3390/nu15112569

**Published:** 2023-05-30

**Authors:** Francina J. Dijk, Zandrie Hofman, Yvette C. Luiking, Matthew J. W. Furber, Justin D. Roberts, Ardy van Helvoort, Miriam van Dijk

**Affiliations:** 1 Danone Nutricia Research, 3584 CT Utrecht, The Netherlands; francina.dijk@danone.com (F.J.D.); zandrie.hofman@danone.com (Z.H.); yvette.luiking@danone.com (Y.C.L.); matthew.furber@danone.com (M.J.W.F.); justin.roberts@danone.com (J.D.R.); ardy.vanhelvoort@danone.com (A.v.H.); 2 Cambridge Centre for Sport and Exercise Sciences, Anglia Ruskin University, Cambridge CB1 1PT, UK; 3 NUTRIM School of Nutrition and Translational Research in Metabolism, Maastricht University Medical Center+, 6211 LK Maastricht, The Netherlands

**Keywords:** ageing, amino acids, casein, arginine, leucine, muscle protein synthesis, pea protein, plant-based protein, soy protein, whey protein

## Abstract

P4, a specific combination of dairy proteins (whey and casein) and plant-based protein isolates (pea and soy), has been shown to provide a more balanced amino acid (AA) profile than its single constituent proteins; however, less is known about how this translates to muscle protein synthesis (MPS). The aim of this study was to investigate the effect of P4 compared to whey or casein against fasted control on MPS. C57BL/6J mice, aged 25 months, were fasted overnight, followed by oral gavage of either whey, P4, casein, or water as a fasted control. Thirty minutes after ingestion, puromycin (0.04 µmol∙g^−1^ bodyweight) was subcutaneously injected; 30-min thereafter, mice were sacrificed. MPS was measured by the SUnSET method, and signalling proteins were determined in the left-tibialis anterior (TA) muscle by the WES technique. AA composition was determined in plasma and right-TA muscle. Dried blood spots (DBS) were analysed for postprandial AA dynamics at 10, 20, 45, 60 min. MPS was 1.6-fold increased with whey (*p* = 0.006) and 1.5-fold with P4 compared to fasted (*p* = 0.008), while no change was seen with casein. This was confirmed by a significant increase of phosphorylated/total ratio of 4E-BP1 for both whey (*p* = 0.012) and P4 (*p* = 0.001). No changes were observed in p70S6K and mTOR phosphorylation/total ratio with whey or P4. Intramuscular leucine levels were lower for P4 (0.71 µmol∙g dry weight^−1^) compared to whey (0.97 µmol∙g dry weight^−1^) (*p* = 0.0007). Ten minutes postprandial, DBS showed significantly increased blood AA levels of BCAAs, histidine, lysine, threonine, arginine, and tyrosine for P4 versus fasted. In conclusion, a hybrid mix of dairy and plant-based proteins (P4) resulted in a MPS response that was similar to whey protein in aged mice after fasting. This suggests that other anabolic triggers beyond leucine or the well-balanced amino acid profile and bioavailability of the blend benefit stimulation of MPS.

## 1. Introduction

The development and regulation of skeletal muscle is the result of a dynamic balance between protein synthesis and breakdown. It is known that with ageing, changes in muscle protein turnover may lead to a decline in skeletal muscle mass, leading to decreased muscle strength and physical function [1,2,3,4]. Historically, much focus has been on dietary supplementation of animal protein sources, and especially whey protein, to attenuate age-induced muscle mass loss by stimulating muscle protein synthesis [5,6]. More recently, plant-based proteins are being explored to function as bioactive and sustainable alternatives [7,8]. Another approach could be blending dairy and plant-based protein sources to enhance protein quality. A combination of dairy proteins (rich in essential amino acids (EAAs [9]); and plant-based proteins (rich in conditionally essential amino acids (CEAA)) could provide a more complete and balanced amino acid (AA) profile with higher amounts of otherwise rate-limiting EAAs and CEAAs [10,11,12]. Increasing the rate-limiting amino acids may prevent excessive transamination and improve the availability of AAs for their metabolic use in the body, such as muscle protein synthesis (MPS), which is specifically important in a state of prolonged fasting and/or increased metabolic stress, found in the aged population, in acute hospitalised frail or critical care patients or patients with chronic disease.

There is current clinical interest in exploring nutritional opportunities to proactively stimulate MPS in critical care patients undergoing tube feeding, to support or improve muscle metabolism and potentially facilitate the recovery process. In an ageing population, it is important to consider the demands on the health care system and to develop nutritional approaches to enhance patient recovery times, reduce hospital duration, and additionally support independence in daily life [13]. Therefore, it is important to investigate and find the most optimal combination of proteins to induce muscle protein synthesis, especially in the context of ageing, critical care, and metabolic stress.

A practical, feasible and translatable in vivo model to study critical care patients is not presently available [14,15]. However, it is possible to model the critical care patient by including ageing in combination with metabolic stress induced by fasting. It is known from aged mice that their anabolic capacity decreases [16] and that this anabolic threshold can be overcome by providing amino acids as building blocks in the form of whey protein enriched with the BCAA leucine to trigger the MPS response [17]. To measure MPS in this model, the SUnSET method was used [18]. This technique uses puromycin, a structural analogue of tyrosyl-tRNA, and anti-puromycin antibodies to detect the amount of puromycin incorporation into newly synthesised peptides. The accumulation of puromycin-coupled peptides accurately reflects the rate of protein synthesis. Goodman et al. made a comparison between the traditionally used incorporation of labelled amino acid method and the SUnSET method and proved that the SUnSET technique is a reliable method for the measurement of in vivo changes in protein synthesis of the muscle [18,19].

The aim of this study was to investigate the effect of a protein blend from different dairy- and plant-based protein sources (whey, casein, soy, and pea), compared to single dairy-based protein sources whey and casein, on MPS in aged mice after fasting. Whey is considered the gold standard for inducing MPS [17,20]. Fasting will ensure that all animals are in the same nutritional state and, moreover, mimic the metabolic stress observed in critical care patients. To further unravel the mechanistic processes of MPS, the mammalian target of rapamycin (mTOR) pathway signalling proteins were explored. In addition, to better understand the relevance of postprandial leucine and other AAs in blood and muscle as a proxy of amino acid bioavailability, we measured plasma and muscle free AA concentrations at one timepoint and dried blood spots (DBS) analyses over time. Finally, to provide first insights into protein digestibility, stomach protein content was also quantified.

## 2. Materials and Methods

### 2.1. Animals

**Figure 1:**
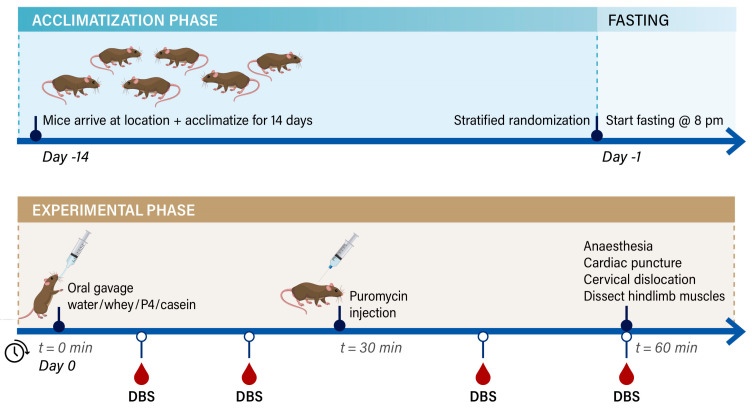
Experimental time schedule of the experiment divided into the acclimatisation, fasting and experimental phase. The time schedule is similar for both cohorts; DBS sampling was only performed in cohort 2.

This study was conducted under an ethical license of the national competent authority (CCD, Centrale Commissie Dierproeven), including positive advice from an external, independent Animal Ethics Committee (St. DEC consult, Soest, The Netherlands), and all animal procedures were captured in a protocol approved by the Animal Welfare Body, also following the principles of good laboratory animal care of Wageningen University—by this process securing full compliance the European Directive 2010/63/EU for the use of animals for scientific purposes (protocol approval code: INUTIN-21-01-01_WNL_MvD). Aged male C57BL/6J mice of 25 months of age were obtained from Janvier Labs (Saint Berthevin, France). Animals (cohort 1 in total: 40 and cohort 2 in total: 32) were individually housed to be aligned with and to compare the data to previous long-term studies where food intake and physical activity were analysed [16,17,21]. Animals were housed in a climate-controlled room (12:12 dark:light cycle from 6:00–18:00 lights on, from 18:00–6:00 lights off) with a constant room temperature of 21 ± 1 °C and humidity of 57 ± 2%). Housing consisted of Makrolon Type III cages (Techniplast, Buguggiate, Italy) with standard bedding (Lignocel, BK8/15 radiated, Tecnilab, Someren, The Netherlands) and tissues (Manutan, Den Dolder, The Netherlands). No other cage enrichment was provided, again to be aligned with the previous long-term studies where physical activity was measured (enrichment material such as tubes/shelters disrupt the infrared lights. Mice had ad libitum access to a standard semi-synthetic diet (AIN93M, Sniff, Germany) and had free access to tap water from a drinking bottle, refreshed twice a week. Upon arrival, mice were allowed to acclimatise for 2 weeks, and body weight was determined twice a week in the afternoon at the end of the light phase. On the day before the section, animals were fasted overnight by taking the food away at 8 pm during the dark phase until 8 am the next morning but still allowed access to water. On the day of the section, mice were stratified and randomised into 4 groups with no greater deviation than 5% from the overall mean body weight. The experiment was executed in 2 subsequent cohorts: Cohort 1 was executed at GDL Utrecht University (Utrecht, The Netherlands) with group size: fasted: n= 4, whey: n= 7, P4: n= 8 and casein n= 9; and cohort 2 was executed at CARUS Wageningen University (Wageningen, The Netherlands) with group size: fasted: n= 3, whey: n= 7, P4: n= 6 and casein n= 7; combing both cohorts resulted in total group numbers of fasted: n= 7, whey: n= 14, P4: n= 14 and casein n= 16 with a drop out due to ageing related symptoms of 7 animals in cohort 1 and 8 animals in cohort 2. For the experimental schedule, see Figure 1. All researchers involved were blinded for the provided protein supplementation until all data was collected.

### 2.2. Experimental Protocol

On the experimental day, mice received an oral gavage (end volume 0.5 mL) containing approximately 70 mg protein (0.75 g∙kg^−1^ body weight). Protein contents of casein and P4 (35% whey, 25% casein, 20% pea and 20% soy) were matched to whey protein content (see Table 1), and an equal amount of tap water was used as a fasted control. The amount of protein provided to the mice mimics an effective amount of 20 g whey protein to stimulate MPS in elderly humans [22], taking into account the daily protein recommendation of humans and mice [23]. Thirty minutes after oral gavage, mice received a s.c. injection with 0.04 mmol∙g^−1^ body weight puromycin (Sigma-Aldrich, Merck, Zwijndrecht, The Netherlands) (Figure 1) [24]. In between animal handling, mice were returned to their home cages, where they were permitted free access to water only. After an additional 30 min, a maximal 1000 µL of blood was drawn by cardiac puncture under total isoflurane anaesthesia (isoflurane/N_2_O/O_2_), after which cervical dislocation was applied. During the postprandial period of cohort 2, 1–2 droplets of blood obtained via tail cut were stamped directly on a protein saver card at 10, 20, 45 and 60 min. Whole blood was collected in heparin-coated tubes (12.8 µL heparin (Heparine Leo 5000 I.E.∙mL^−1^ from a local Pharmacia, The Netherlands) and 7.2 µL of PBS). Plasma samples were prepared by centrifugation and collecting the plasma for amino acid analyses. Finally, MPS was measured after a 60 min postprandial period (SUnSET method [20]). The muscles from the hind limb were excised, weighed, snap-frozen in liquid nitrogen and stored at −80 °C until combined analyses. A schematic illustration of the animal experiment is shown in Figure 1. 

### 2.3. Protein Simple Western^TM^ Analysis

Left tibialis anterior muscles (TA) were cut into pieces and homogenisation buffer (40 mM Tris pH 7.5; 1 mM EDTA; 5 mM EGTA; 0.25% Triton X-100; Pierce Protease and Phosphatase Inhibitor Mini Tablets, EDTA free (Thermo Fisher, Landsmeer, The Netherlands) (1 tablet per 10 mL buffer) was added in a ratio of 10 µL∙mg^−1^ muscle. Muscles were homogenised using a FastPrep-24 (MP Biomedicals, Eindhoven, The Netherlands) with Precellys® Ceramic kit tubes (1.4/2.8 mm ceramic mixture) (VWR, Amsterdam, The Netherlands) and homogenised 3 times for 30 s on 6.5 m∙s^−1^ speed. Subsequently, samples were placed at 4 °C for 1 h with continuous shaking followed by one more run in the FastPrep-24. The homogenates were removed from the tubes with ceramic beads and brought to clean tubes. Protein content was determined using the BCA protein assay kit (Pierce, Thermo Fisher, Landsmeer, The Netherlands). Puromycin incorporation into proteins to determine MPS was measured by Simple Western^TM^ analysis using Wes™ (ProteinSimple, a Bio-Techne brand, San Jose, CA, USA); briefly, 0.5 g∙L^−1^ muscle homogenate with 20% Fluorescent Master Mix was loaded onto the WES plate after heat treatment for 5 min at 100 °C, using the 12–230 kDa Separation Module (ProteinSimple). Five times diluted anti-puromycin (3RH11, Kerafast, Boston, MA, USA) was loaded on the WES plate, and Anti-mouse Detection Module for Wes™ (ProteinSimple) was used as a secondary antibody and detection. The run was performed using 30 min separation time, 375 V separation voltage, 30 min antibody diluent time, 30 min primary antibody time and 30 min secondary antibody time. The same sample was used to measure total protein content with the Total Protein Detection Module for WES (ProteinSimple), with Wes™ settings: 30 min separation time, 375 V separation voltage, 30 min Biotin Labeling Time, 30 min Antibody Diluent Time and 30 min Total Protein HRP Time. Samples of each group were equally distributed over the Wes™ runs so that each run contained all different groups. To compare different Wes™ runs, a pooled sample of all fasted samples was created and taken along each WES run and used to correct for differences between Wes™ runs. 

The total area under the curve of puromycin and total protein run were calculated by the Compass for SW software version 6.1.0 (ProteinSimple), with ratios to the fasted group calculated as a measure for puromycin incorporation and total protein. MPS was calculated by dividing puromycin by total protein. mTOR pathway proteins were determined with the Wes™ using the same settings as for MPS. The following antibodies were used: anti-4EBP1 (9452, Cell Signaling Technology, 5× diluted) and anti-phospho-4EBP1 (Thr37/46) (2855, Cell Signaling Technology, 50× diluted), anti-p70S6k (34475, Cell Signaling Technology, 500× diluted), anti-phospho-p70S6k (Thr421/Ser424) (9204, Cell Signaling Technology, 10× diluted), anti-mTOR (2983, Cell Signaling Technology, 10× diluted) and anti-phospho-mTOR (Ser2448) (2971, Cell Signaling Technology, 10× diluted). For 4EBP1 and p70S6k antibodies, the 12–230 kDa Separation Module (ProteinSimple) was used; for mTOR antibodies, the 66–440 kDa Separation Module (ProteinSimple) was used. For detection, Anti-Rabbit Detection Module (ProteinSimple) was used. The total peak area was calculated by the Compass for SW software, with ratios to the fasted group also calculated for each antibody. 

### 2.4. Biochemical Measurements

In plasma and TA muscle, endpoint (t = 60 min) amino acid concentrations were measured using ultra-fast liquid chromatography (UFLC) [25]. Right TA muscle was freeze-dried, homogenised in 2% perchloric acid, centrifuged (2000× *g* for 20 min at 4 °C), and supernatants were used to determine muscle free amino acid concentrations. The bioavailability of the amino acids for muscle is relevant to the muscle protein synthesis data; therefore, amino acid profiles were determined from DBS. DBS were analysed by Nutrition Analytical Service at the Institute of Aquaculture (University of Stirling, Stirling, UK). Briefly, a single 5 mm disc was punched out of the DBS (where DBS were very small, the punch was positioned to ensure as much blood coverage of the spot as possible). The dried blood was extracted from the spot in 0.1% sodium dodecyl sulphate (SDS) and methanol at 37 °C for 30 min. Following evaporation of the methanol, the samples were resuspended in acetonitrile: ultra-pure water (1:1) before derivatisation using the Waters AccQ-Tag Ultra Derivatization kit. Amino acid profiles were determined by UPLC instrumentation using the Waters UPLC® Amino Acid Analysis (AAA Solution) on a Waters H-Class UPLC and an Acquity BEH Phenyl 1.7u UPLC column. The DBS data can be used as a tool to investigate amino acid dynamics; however, due to different analysis methods, it cannot directly be compared to the plasma or intramuscular amino acid concentrations in this experiment. 

Amino acid data were grouped in BCAA (isoleucine, leucine and valine), EAA (isoleucine, leucine and valine, histidine, lysine, methionine, phenylalanine, threonine and tryptophan), CEAA (arginine, glutamine, glycine, serine and tyrosine), and NEAA (alanine, arginine, asparagine, aspartic acid, citrulline, glutamine, glutamic acid, glycine, serine and tyrosine). All mentioned amino acids were L-amino acids.

### 2.5. Stomach Content

Since, in contrast to casein, whey and P4 are known for their non-coagulating properties [26], we investigated the content of the stomachs of cohort 1 of the experiment. The complete stomachs, including the content of the first cohort of the experiment, were dissected and weighed. After being cut open dorsally, a photo was taken, and the content was scraped out and weighed. All were collected and frozen at −80 °C until further use. The protein content of the scrapes was analysed using the BCA method.

### 2.6. Statistical Analysis

To determine if the datasets of the 2 cohorts of the experiment could be combined, the fasted plasma leucine levels of both cohorts were analysed to assess whether the basal conditions were similar to prior (unpublished) studies [16,17] and statistical equivalence was achieved. Therefore, fasted samples of 5 previously performed independent experiments were analysed. This resulted in a plasma leucine range from a lower limit of 125.8 to an upper limit of 303.4 µmol∙L^−1^, a biological variance of 177.5 µmol∙L^−1^. The overall plasma leucine average was 202.0 ± 37.4 µmol∙L^−1^. Since the leucine plasma values of cohort 2 were within the two-sided 90% confidence interval (150–250 µmol∙L^−1^), it was deemed acceptable to combine the complete data set for all analyses.

For sample size calculations, the primary outcome parameter is MPS. MPS data were normally distributed and tested with Shapiro–Wilk Test for Normality and equal variance. MPS data were tested for outliers using the Grubbs outlier test with α = 0.05, resulting in 1 outlier in the fasted group of cohort 1 and 1 outlier in the fasted group of cohort 2. Sample size calculations for the first cohort were based on the comparison of fasted (MPS ratio 1.080) vs whey (MPS ratio 1.550) groups. The power was set to 80%, variation was 2.9, α of 0.01, resulting in 8 animals per group; taking into account a drop out of 10%, 8 additional animals were requested. For cohort 2, sample size calculations were improved by including more comparisons and testing superiority. It was hypothesised that the P4 group was superior to the whey and casein group, and to reduce the number of animals, our approach was to combine with the data of cohort 1. MPS ratio means used for calculations: fasted: 1.00 ± 0.24, whey: 1.419 ± 0.31, P4: 1.763 ± 0.34 and for casein: 1.378 ± 0.41 (mean ± std). Following the O’Brien–Fleming approach for 1 interim (cohort 1) and final analyses (including cohort 2), α was one-sided 0.0492 / 2 + 0.0246. This resulted in a total number of animals of fasted n = 5, whey n = 16, P4 n = 16 and casein n = 16, with a power of 80%.

Statistical analyses were performed using GraphPad Prism version 8.0.0 for Windows (GraphPad Software, San Diego, CA, USA). One-way ANOVA followed by LSD post hoc analysis was used to compare the difference between supplemental groups. The area under the curve of individual DBS curves were calculated with the area under the curve analysis of GraphPad Prism. In the setting of the experiment, it was not possible to take DBS samples at t = 0 min; therefore, amino acid values of t = 10 min of the fasted group were used as t = 0 min values for each group, and this was used as a baseline in AUC calculations. All data are expressed as means ± SEM. Statistical significance was defined as *p* < 0.05. 

## 3. Results

No significant differences in body weight, liver and individual hind limb muscles wet weights were found within and between the two different cohorts, demonstrating groups were randomised well and confirming that it is justified to combine results of both cohorts. Nutritional supplementations were analysed on amino acid content and confirmed that equal amounts of protein supplementation were provided in both cohorts. Table 1 shows the average results of both cohorts; note that not all amino acids were measured due to analytical constraints, and variation between preparations caused small differences between the two different cohorts.

### 3.1. In Vivo Muscle Protein Synthesis

**Figure 2:**
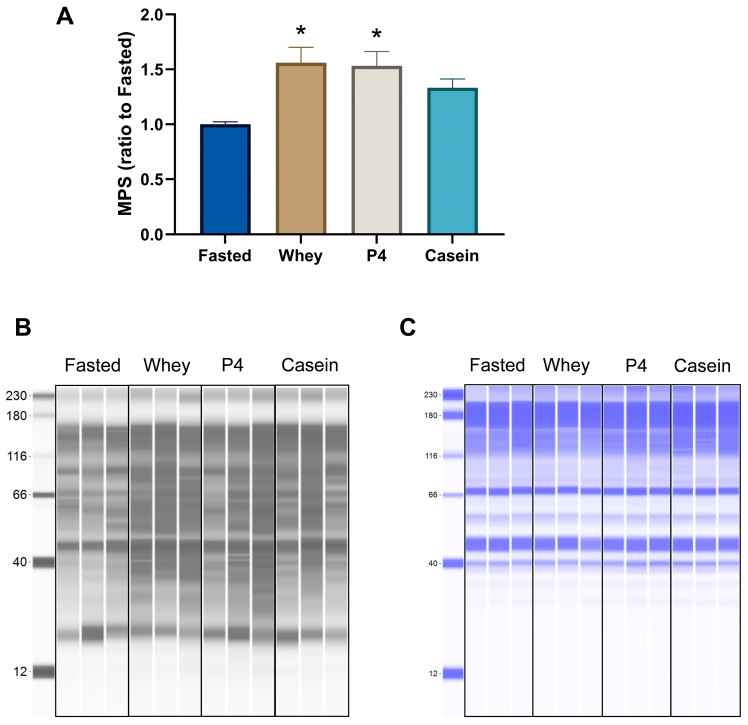
Muscle protein synthesis assessed by the SUnSET method and measured in the TA after overnight fasting (Fasted) or supplementation with whey, P4 or casein. (**A**) MPS ratio of puromycin-labelled peptides, (**B**) WES result of puromycin-labelled proteins of three representative samples of each group. (**C**) WES result of total protein of three representative samples of each group showing equal loading of samples. Values are means ± SEM and are expressed as a relative ratio to the fasted control group. * *p* < 0.05 compared to the fasted control group.

**Figure 3:**
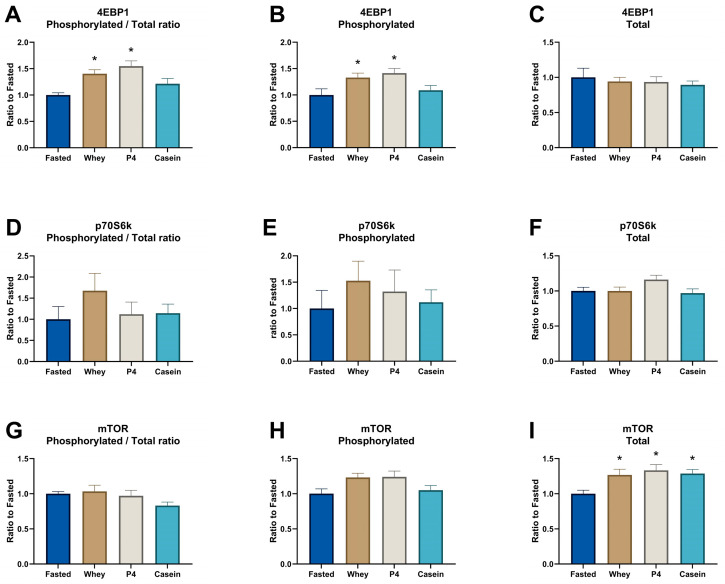
Signal transduction proteins involved in the protein machinery in the TA after overnight fasting (Fasted) or supplementation with whey, P4 or casein. (**A**) 4EBP1 ratio between phosphorylated and total 4EBP1, (**B**) phosphorylated protein of 4EBP1, (**C**) total protein of 4EBP1, (**D**) p70S6K ratio between phosphorylated and total p70S6K, (**E**) phosphorylated protein of p70S6K, (**F**) total protein of p70S6K, (**G**) mTOR ratio between phosphorylated and total mTOR, (**H**) phosphorylated protein of mTOR, and (**I**) total protein of mTOR. Values are means ± SEM of both experiments and are expressed as a relative ratio to the fasted control group. * *p* < 0.05 compared to the fasted control group.

Supplementation of whey and the P4 protein blend induced a similar significant MPS increase compared to the fasted control group after prolonged fasting (*p* = 0.005 for whey and *p* = 0.008 for P4, Figure 2A). Equal amounts of casein did not show a significant MPS response after 1 h of supplementation (*p* = 0.085 vs fasted). In line with the MPS response, supplementation of whey and P4 resulted in a significantly increased phosphorylated/total ratio of 4EBP1 compared to fasted control (*p* = 0.012 for whey and *p* = 0.001 for P4) (Figure 3A). Phosphorylated 4EBP1 was also significantly changed for whey (*p* = 0.04) and P4 (*p* = 0.011) (Figure 3B); the total 4EBP1 was not changed (Figure 3C). p70S6K phosphorylated/total ratio was not significantly increased, although supplementation with whey was 1.7 times higher compared to fasted (*p* = 0.207) (Figure 3D). Phosphorylated and total p70S6k were not changed (Figure 3E,F). The phosphorylated/total ratio of mTOR showed no differences with all supplementations (Figure 3G). Interestingly, phosphorylated mTOR was not changed after whey, P4 and casein supplementation (Figure 3H), but total mTOR was significantly increased after all protein supplementations compared to fasted (*p* < 0.036) (Figure 3I).

### 3.2. Amino Acid Dynamics from Dried Blot Spots

**Figure 4:**
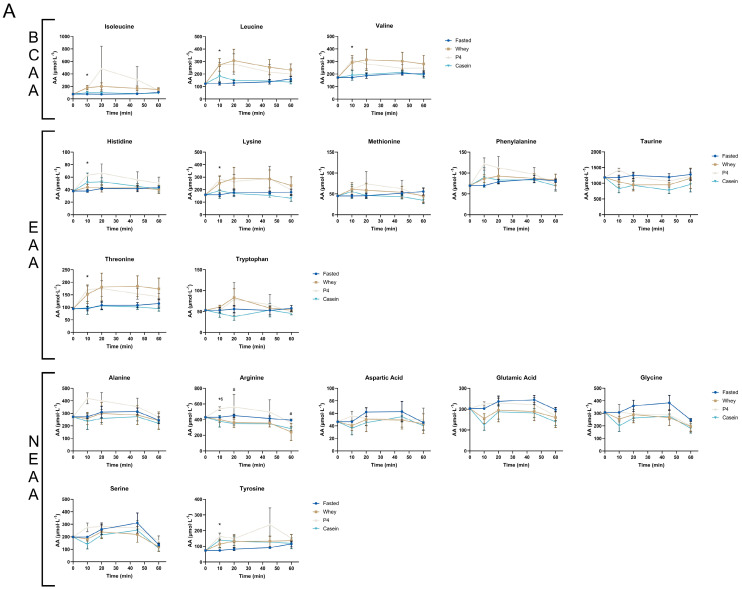

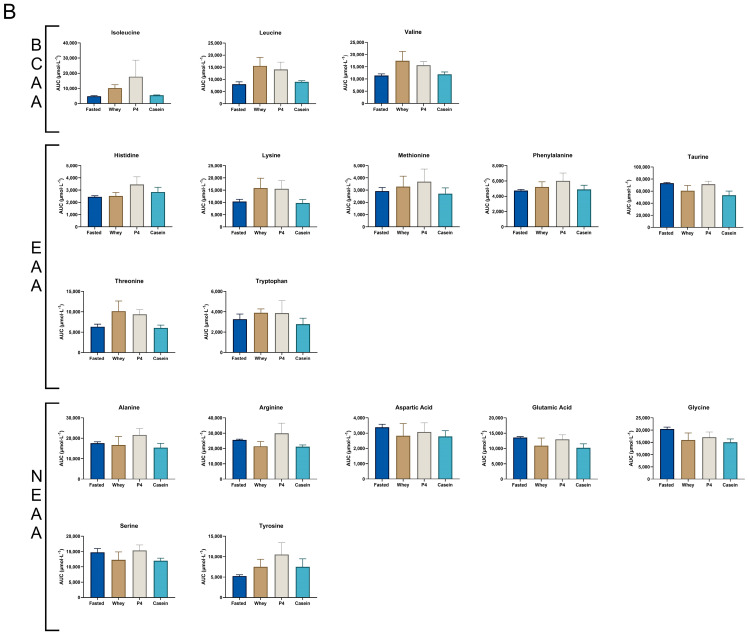
Dried Blood Spot amino acid analyses at 10, 20, 45 and 60 min post protein supplementation. (**A**) Time curve of individual DBS amino acids. (**B**) AUC of individual DBS amino acids. Values are means ± SEM of samples of cohort 2. In time curves, * *p* < 0.05 of P4 vs fasted, # *p* < 0.05 of casein vs F, $ *p* < 0.05 of P4 vs whey.

DBS leucine levels increased equally and quickly 10 min after both whey (273.0 µmol∙L^−1^) and P4 (276.8 µmol∙L^−1^) supplementation (Figure 4A). Interestingly, the increase in leucine at 10 min was only significant following P4 (*p* = 0.003) but not for whey (*p* = 0.089). Leucine peaked at 20 min after supplementation and decreased thereafter. Casein supplementation did not significantly increase plasma leucine levels in the analysed time frame. At 10 min after P4 supplementation, blood amino acids of isoleucine, valine, histidine, lysine, threonine, arginine, and tyrosine were also significantly increased compared to the fasted condition (for all *p* < 0.050), but not after whey or casein supplementation. After 20 and 60 min of casein supplementation, blood arginine levels were significantly decreased compared to fasted (*p* = 0.020, *p* = 0.047, respectively). Other measured AAs were not significantly changed at specific time points (Figure 4A). Calculated AUC of DBS did not result in any significant differences between the supplemental groups compared to fasted for all AAs measured (Figure 4B).

### 3.3. Amino Acid Concentrations in Plasma at the Endpoint

**Figure 5:**
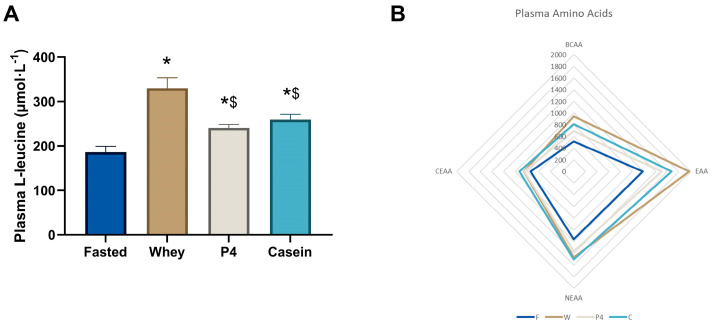

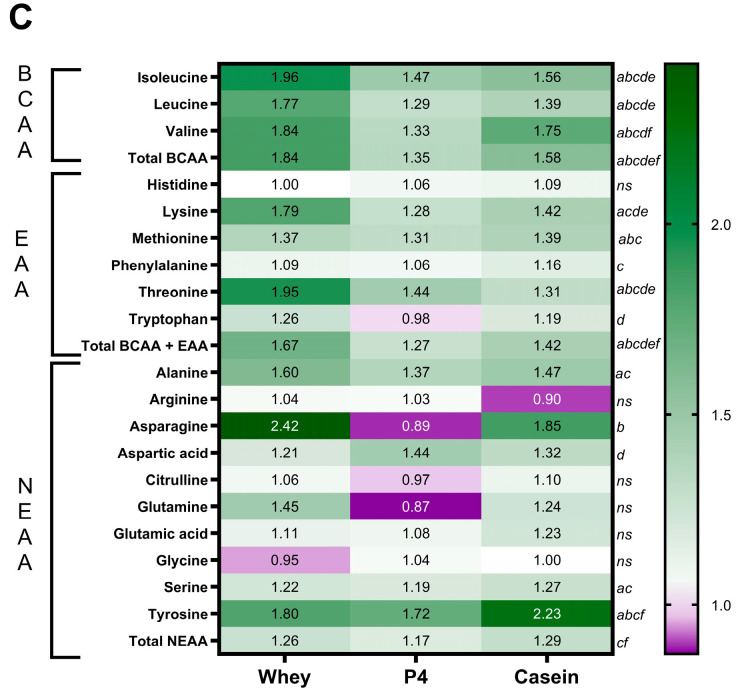
Postprandial amino acid concentrations in plasma. (**A**) Plasma leucine concentrations at 60 min. Values are means ± SEM, * *p* < 0.05 compared to fasted; $ *p* < 0.05 compared to whey. (**B**) Radar plot of plasma BCAA, EAA, CEAA and NEAA concentrations. (**C**) Heatmap of plasma amino acid ratios compared to the fasted group. Significances in the heatmap (*p* < 0.05) are shown by a: fasted to whey, b: fasted to P4, c: fasted to casein, d: whey to P4, e: whey to casein and f: P4 to casein, ns is not significant, as indicated next to the casein column.

Plasma leucine levels at 60 min, as shown in Figure 5A, were significantly increased after supplementation with whey (1.8-fold increase, *p* < 0.0001), P4 (1.3-fold increase, *p* = 0.0401) and casein (1.4-fold increase, *p* = 0.006) compared to fasted, however with whey the increase was significantly higher than with P4 or casein (*p* < 0.001) (Figure 5A). The complete dataset is available in Appendix A Table A1.

Figure 5B illustrates the sum of the different AA groups in plasma (BCAA, EAA, CEAA and NEAA), where fasted shows the lowest content, followed by P4, casein and whey with the highest content. As demonstrated in the heat map in Figure 5C, many other amino acids are differentially present in the plasma after protein supplementation. After 60 min of supplementation, mice fed with whey showed significantly elevated levels of leucine, isoleucine, valine, lysine, methionine, threonine, alanine, serine, and tyrosine, compared to fasted (all *p* < 0.05). P4 showed significant increases of isoleucine, leucine, valine, methionine, threonine, aspartic acid, and tyrosine (all *p* < 0.05 vs fasted), while casein showed significant increases of isoleucine, leucine, valine, lysine, methionine, phenylalanine, threonine, alanine, serine, and tyrosine (all *p* < 0.05 vs fasted). Plasma BCAA levels were significantly increased by 1.84 times compared to fasted after whey supplementation (*p* < 0.0001), 1.35 times compared to fasted after P4 supplementation (*p* = 0.020), and 1.58 times compared to fasted after casein supplementation (*p* = 0.0001). Plasma EAA levels were significantly increased compared to fasted following whey (1.67 times, *p* < 0.0001), P4 (1.27 times, *p* = 0.003) and casein supplementation (1.42 times, *p* < 0.0001). Total plasma NEAA levels were increased 1.26 times compared to fasted after whey supplementation (*p* = 0.009) and 1.29 times after casein supplementation (*p* = 0.003) (Figure 5C), but not with P4 (*p* = 0.077). For statistical differences, the different groups were compared for individual amino acids with one-way ANOVA; therefore, multiple comparison analysis was not needed in this experiment.

### 3.4. Free Amino Acid Concentrations in TA Muscle at the Endpoint

**Figure 6:**
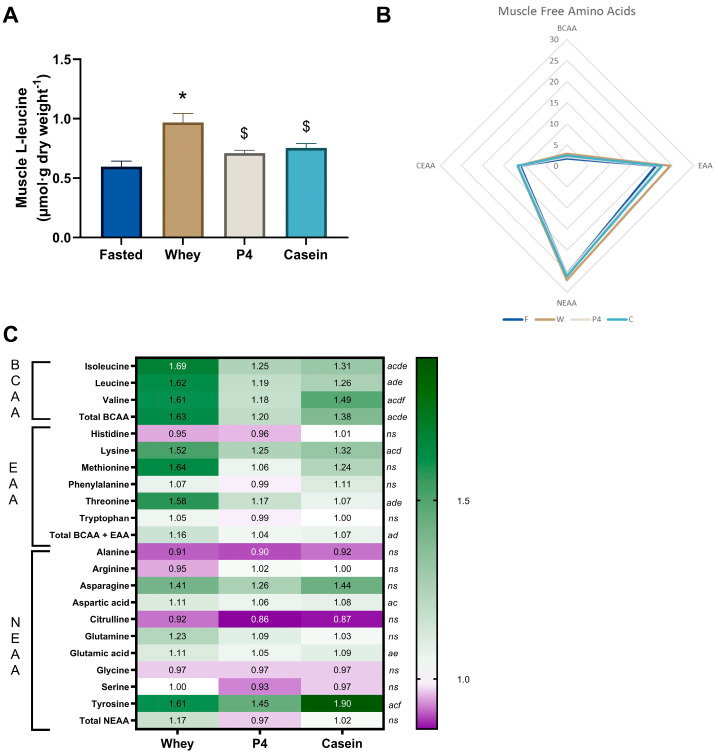
Postprandial free amino acid concentrations in muscle at 60 min. (**A**) Muscle free leucine concentrations. Values are means ± SEM, * *p* < 0.05 compared to fasted; $ *p* < 0.05 compared to whey; (**B**) Radar plot of muscle free BCAA, EAA, CEAA and NEAA concentrations. (**C**) Heatmap of muscle free amino acid ratios compared to the fasted group. Significances in the heatmap (*p* < 0.05) are shown by a: fasted to whey, b: fasted to P4, c: fasted to casein, d: whey to P4, e: whey to casein and f: P4 to casein, ns is not significant, as indicated next to the casein column.

As shown in Figure 6A, muscle free leucine was significantly increased after whey supplementation (*p* < 0.0001); however, after P4 and casein supplementation muscle free leucine levels were not significantly elevated. Similar to the plasma AA profiles, Figure 6B shows the sum of the different AA groups in muscle, indicating small differences between the treatment conditions. As shown in the heatmap in Figure 6C, supplementation of whey showed significant increases of isoleucine, leucine, valine, lysine, threonine, glutamine, and tyrosine compared to fasted (all *p* < 0.05). P4 showed no significant differences in amino acids compared to fasted, while casein showed significant increases in isoleucine, leucine, valine, lysine, aspartic acid, and tyrosine (all *p* < 0.05). Muscle BCAA levels were significantly increased compared to fasted with whey (1.63 times, *p* < 0.0001) and casein (1.38 times, *p* = 0.006), but not with P4 (*p* = 0.153), whereas muscle free EAAs were only significantly increased with whey supplementation (1.16 times, *p* = 0.016) (Figure 5C). The complete dataset is available in Appendix A Table A2.

### 3.5. Stomach Content

**Figure 7:**
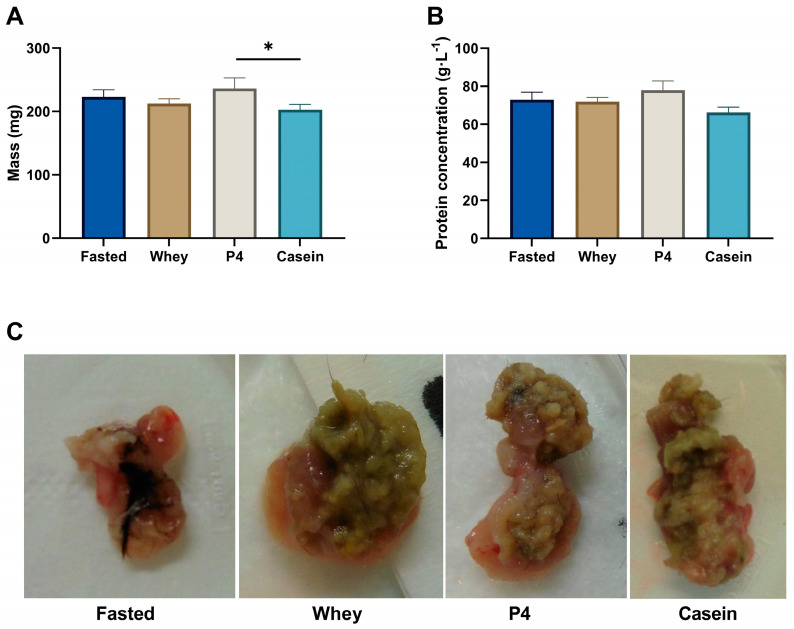
Stomach characteristics after overnight fasting (Fasted) or 60 min after supplementation with whey, P4 or casein. (**A**) weight of the complete wet stomach, (**B**) protein content of the stomach scrapes and (**C**) representative photographs of the stomach: fasted mouse 6, whey mouse 11, P4 mouse 20 and casein mouse 45. Values are means ± SEM of cohort 1. * *p* < 0.05.

The weight of the complete stomach after 60 min of supplementation did not show any differences between the different protein sources and the fasted state (Figure 7A). The photographs of the opened stomach depict that there is still content present after prolonged fasting (Figure 7C, fasted). Some fasted animals had blood, fur/ hairs, or faeces in the stomach (four out of eight mice). However, no difference in protein content of the stomach scrapes was found between the supplements compared to the fasted condition (Figure 7B), suggesting all protein supplementation had passed the stomach and entered the gut-intestinal tract. Interestingly, there is a significant difference in stomach weight between P4 and casein supplementation (*p* = 0.041). The morphology of the stomach after protein supplementation appeared different compared to the fasted group (Figure 7C).

## 4. Discussion

To our knowledge, this is the first experiment to investigate the MPS response of a hybrid blend of dairy (whey, casein) and plant-based (pea, soy) proteins (P4), compared to single dairy protein sources in aged mice undergoing prolonged fasting. P4 and whey protein stimulated MPS to a similar level compared to the fasted control in this model despite differences in the AA content of protein sources and differences in AA content measured in plasma, muscle, or DBS. Casein did not show a significant increase in MPS. Both whey and P4 activate the mTOR pathway via phosphorylation of 4EBP1.

There were other papers studying the effect of a dairy-plant blend that showed similar results on MPS. Borack et al. [27] studied the effect of 30 g protein from a soy-dairy (soy, whey, casein) blend compared to whey protein isolate on protein synthesis responses in older men. However, this study tested the protein intervention in combination with exercise. The study showed similar responses on MPS and mTORC1 signalling for the soy-dairy blend and whey protein isolate. Butteiger et al. [28] studied the effect of a meal containing soy, whey, and caseinate blend compared to whey protein and soy protein isolate on skeletal MPS in rats using two different ratios of the blend. However, this was not an acute model and proteins were given in a complete meal. The data showed that both the blends, as well as the single sources of protein, enhance skeletal MPS. Other papers studied the effect of a plant-based protein blend compared to dairy protein, Pinckaers et al. [29] compared a blend of wheat, corn and pea protein to milk protein in healthy young males. The data showed no difference in MPS rates between the plant-based protein blend and the dairy protein. The same authors also showed no difference in MPS with a blend of wheat and milk protein compared to the same amount of milk protein or wheat protein in healthy young males [30]. This might be different in the elderly population due to increased anabolic resistance [17].

In line with our findings, previous studies comparing whey with casein have shown differences in MPS; therefore, it is expected that the MPS of whey is higher compared to casein [20,31]. Soy protein also showed a lower MPS response compared to whey protein, as previously shown by Tang et al. [20] and Yang et al. [32]. 

In all these papers, it is shown that the anabolic amino acid, leucine, is the main driver of MPS, similar to our current findings and from a previous study [17]. A balanced amino acid profile obtained by blending different protein sources lowers the risk of a lack of specific amino acids versus a single source and contributes to a sustained MPS response. Moreover, in the aged population, it is important to provide enough protein to overcome anabolic resistance. 

In our study, the results of the P4-MPS response may be explained by the fact that P4, besides having a good protein quality similar to whey, has a more balanced amino acid profile resulting in increased BCAAs, EAAs histidine, lysine threonine, CEAA arginine and NEAA tyrosine as shown by DBS results in the early postprandial phase. This may increase the systemic amino acid availability for MPS after splanchnic extraction by reducing the amount of transamination during the metabolic stress of prolonged fasting. P4 contains less leucine, BCAAs and EAAs compared to whey protein and casein but does have more arginine, as shown in Table 1. DBS data also showed that arginine levels after P4 supplementation were increased 10 min after ingestion, in contrast to whey. Arginine is considered to be a CEAA, meaning that under specific conditions, arginine is essential. This may have pertinent implications as the mice in the current experiment were under metabolic stress due to the prolonged fasting state. It is very well possible that under this condition, MPS may be stimulated with leucine but supported via arginine in activating the mTOR pathway via NO [33,34]. 

The P4 blend is a mix of dairy and plant-based protein sources used in tube feeding for medical applications in patients. This hybrid protein blend, which contains significant amounts of the anabolic amino acid leucine, has been developed to have a well-balanced amino acid pattern of essential and conditionally essential amino acids, as in contrast to dairy proteins, plant-based proteins have relatively high amounts of the conditional amino acids arginine and glycine [10]. These conditional essential amino acids play an important role in protein kinetics and MPS and can become limiting in acute critical care or chronic diseases [35]. Moreover, arginine supplementation has been shown to have many beneficial effects on disease beyond supporting MPS. Hence, the use of a blend of dairy proteins with plant-based proteins rich in arginine may be a preferred protein combination for patients with metabolic stress and chronic disease [8,36]. The abilities of the P4 blend to support muscle mass and function had not been previously explored. In this study, we demonstrate that P4 is as good as whey in stimulating MPS, supported by the phosphorylation of the downstream signal transduction protein 4EBP1. 

The clinical relevance of our data lies in the possibility of substituting total whey-based products with the P4 blend, thereby offering a flexitarian, more sustainable solution to increase MPS. P4 might also support critical care patients to preserve muscle in their patient journey and to gain muscle function during their recovery. However, the preferred protein source also depends on age, exercise, health status and personal preference (i.e., vegan, vegetarian, intolerances) [7].

In this study, we compare a blend of whey, casein, soy and pea protein to single whey and casein protein but not to a single pea or soy protein. It would have been better to also have added single pea and soy protein as individual groups to be able to compare the effect of all single protein sources with the combination.

In cohort 1, only endpoint parameters were included for analysing AA. A new tool has been implemented in cohort 2, the dried blood spots, however, with a small sample size of 3 animals per group. Dried blood spots provide insights into the amino acid dynamics compared to endpoint analysis, as shown in our results. However, the differences in the analytical methods and difference in absolute values make it difficult to directly compare outcomes of plasma AAs with DBS results, as shown by differences of some AAs at 60 min. This might be explained by the matrix of whole blood on a protein saver card versus directly in plasma. Furthermore, it is still not possible to measure muscle AA dynamics without substantially increasing animal numbers, which is not preferred. 

In preparing this study, it was unclear if aged mice digest slow (casein) and fast proteins (whey) in a similar manner to humans [37]. In the first cohort of this study, we observed that the protein content of the stomach was equally absent for all supplements, suggesting that the content of all supplemented proteins entered the gastrointestinal tract. DBS confirmed that the AAs entered the bloodstream already 10 min after ingestion. By measuring the protein content of the stomach, we aimed to generate insights into protein gastric emptying, as it has been proposed that gastric passage of casein proteins is slower than for whey protein [26].

## 5. Conclusions

In conclusion, a mix of dairy and plant-based proteins (P4) resulted in a MPS response that was similar to whey protein in aged mice undergoing fasting. This suggests that other anabolic triggers beyond leucine or the balanced amino acid profile and bioavailability of the blend stimulate MPS. These findings may have pertinent applications within patients undergoing metabolic stress, like in critical care and frail ageing, where muscle mass and function may be compromised.

## Figures and Tables

**Table 1:** Provided protein supplementations and amino acid contents for each group. Supplementations were prepared fresh at room temperature in tap water and stored at 4 °C until use. The tap water used was also tested but showed all 0 values. Raw material and total protein were equal in both cohorts of the experiment, although made fresh for each cohort. Values of protein content are single measurements for each cohort. Values of amino acid concentrations are mean ± STD of single measurements of cohort 1 + cohort 2.

	Whey	P4	Casein
Raw material and protein content of gavage (mg/0.5 mL)			
Whey (raw material)	83.9	28.6	
Pea (raw material)		15.9	
Soy (raw material)		14.7	
Casein (raw material)		19.0	75.8
Total protein	69.6	67.8	67.6
Measured L-amino acid concentration (mg∙g^−1^ gavage)			
Isoleucine	4.20 ± 0.00	3.15 ± 0.21	3.15 ± 0.07
Leucine	7.15 ± 0.21	5.7 ± 0.57	6.15 ± 0.07
Valine	3.85 ± 0.07	3.35 ± 0.35	4.2 ± 0.00
**Sum BCAA**	**15.2 ± 0.28**	**12.20 ± 1.13**	**13.5 ± 0.00**
Histidine	1.15 ± 0.07	1.30 ± 0.14	1.70 ± 0.00
Lysine	6.15 ± 0.35	4.75 ± 0.49	5.05 ± 0.07
Methionine	1.40 ± 0.14	1.15 ± 0.21	1.80 ± 0.00
Phenylalanine	2.15 ± 0.07	2.80 ± 0.28	3.40 ± 0.00
Threonine	5.15 ± 0.35	3.25 ± 0.49	2.85 ± 0.21
**Sum EAA**	**31.20 ± 1.27**	**25.45 ± 2.62**	**28.30 ± 0.42**
Alanine	3.65 ± 0.21	2.75 ± 0.35	2.05 ± 0.07
Arginine	1.95 ± 0.35	3.10 ± 0.42	2.45 ± 0.07
Asparagine + Aspartic acid	8.00 ± 0.42	6.55 ± 0.78	4.85 ± 0.21
Glutamine + Glutamic acid	12.55 ± 0.49	11.95 ± 1.20	14.90 ± 0.42
Glycine	1.25 ± 0.07	1.70 ± 0.14	1.20 ± 0.00
Serine	3.85 ± 0.21	3.55 ± 0.35	3.95 ± 0.21
Tyrosine	2.10 ± 0.14	2.50 ± 0.28	3.80 ± 0.14
**Sum NEAA**	**33.35 ± 1.91**	**32.10 ± 3.54**	**33.20 ± 1.13**
**Total AA**	**64.55 ± 3.18**	**57.55 ± 6.15**	**61.5 ± 1.56**

## Data Availability

The data presented in this study are available on reasonable request from the corresponding author.

## Appendix A

**Table A1:** Plasma L-amino acid concentrations. Data are mean ± SEM of both experiments. Significances (*p* < 0.05) are shown by a: Fasted to Whey, b: Fasted to P4, c: Fasted to Casein, d: Whey to P4, e: Whey to Casein and f: P4 to Casein, ns is not significant.

L-Amino Acids (µmol∙L^−1^)	Fasted	Whey	P4	Casein
Isoleucine	101.5 ± 5.8	199.3 ± 12.8 ^a^	149.3 ± 4.7 ^bd^	158.4 ± 6.5 ^ce^
Leucine	186.2 ± 13.4	330.2 ± 23.2 ^a^	241.0 ± 7.5 ^bd^	259.6 ± 11.5 ^ce^
Valine	223.0 ± 19.1	410.8 ± 23.3 ^a^	296.7 ± 8.2 ^bd^	390.8 ± 25.3 ^cf^
**Sum BCAA**	**511 ± 37**	**940 ± 58 ^a^**	**687 ± 19 ^bd^**	**809 ± 43 ^cef^**
Histidine	69.4 ± 3.5	69.3 ± 2.7	73.5 ± 2.9	76.0 ± 3.3
Lysine	199.3 ± 13.2	357.4 ± 20.4 ^a^	255.4 ± 14.1 ^d^	283.1 ± 17.0 ^ce^
Methionine	50.9 ± 3.9	69.9 ± 3.4 ^a^	66.5 ± 2.6 ^b^	70.8 ± 4.9 ^c^
Phenylalanine	96.3 ± 4.6	105.3 ± 3.6	101.9 ± 3.6	112.1 ± 4.4 ^c^
Threonine	153.6 ± 11.7	300.0 ± 13.1 ^a^	220.8 ± 10.6 ^bd^	201.9 ± 9.3 ^ce^
Tryptophan	103.5 ± 6.5	130.8 ± 10.1	101.8 ± 8.1 ^d^	122.7 ± 6.9
**Sum EAA**	**1184 ± 60**	**1973 ± 83 ^a^**	**1507 ± 39 ^bd^**	**1676 ± 55 ^cef^**
Alanine	246.9 ± 18.1	396.3 ± 28.3 ^a^	338.6 ± 22.1	362.8 ± 31.1 ^c^
Arginine	63.0 ± 4.8	65.5 ± 7.8	65.1 ± 6.2	57.0 ± 6.9
Asparagine	42.8 ± 2.7	51.7 ± 5.9	61.7 ± 3.4 ^b^	56.2 ± 4.2
Aspartic acid	8.3 ± 1.3	20.2 ± 6.6	7.4 ± 1.0 ^d^	15.5 ± 4.6
Citrulline	71.6 ± 13.4	75.8 ± 8.0	69.1 ± 8.1	78.7 ± 10.2
Glutamine	360.8 ± 18.9	400.4 ± 15.4	387.9 ± 23.2	443.6 ± 32.0
Glutamic acid	50.9 ± 8.5	73.7 ± 15.6	44.4 ± 5.5	63.0 ± 9.1
Glycine	153.4 ± 15.1	146.4 ± 9.2	158.9 ± 8.9	153.7 ± 10.2
Serine	96.8 ± 6.3	117.7 ± 4.4 ^a^	115.6 ± 3.9	123.2 ± 7.7 ^c^
Taurine	704.4 ± 82.1	794.9 ± 80.7 ^a^	560.7 ± 51.4 ^bd^	868.2 ± 121.4 ^c^
Tyrosine	67.5 ± 6.2	121.4 ± 7.5	116.1 ± 7.7	150.3 ± 18.0 ^cf^
**Sum NEAA**	**1162 ± 53**	**1973 ± 83**	**1365 ± 44 ^d^**	**1504 ± 89 ^cf^**
**Sum CEAA**	**742 ± 31**	**851 ± 18**	**844 ± 23**	**928 ± 56 ^c^**
**Total AA**	**2346 ± 96**	**3442 ± 115 ^a^**	**2872 ± 72 ^bd^**	**3180 ± 120 ^cf^**

**Table A2:** Muscle free L-amino acid concentrations. Data are mean ± SEM of both experiments. Significances (*p* < 0.05) are shown by a: Fasted to Whey, b: Fasted to P4, c: Fasted to Casein, d: Whey to P4, e: Whey to Casein and f: P4 to Casein, ns is not significant.

L-Amino Acids (µmol∙g^−1^ Dry Weight)	Fasted	Whey	P4	Casein
Isoleucine	0.36 ± 0.02	0.60 ± 0.04 ^a^	0.44 ± 0.02 ^d^	0.47 ± 0.02 ^ce^
Leucine	0.60 ± 0.05	0.97 ± 0.07 ^a^	0.71 ± 0.02 ^d^	0.75 ± 0.04 ^e^
Valine	0.81 ± 0.06	1.31 ± 0.07 ^a^	0.96 ± 0.04 ^d^	1.21 ± 0.08 ^cf^
**Sum BCAA**	**1.8 ± 0.1**	**2.9 ± 0.2 ^a^**	**2.1 ± 0.1 ^d^**	**2.4 ± 0.1 ^ce^**
Histidine	0.62 ± 0.01	0.59 ± 0.02	0.60 ± 0.02	0.62 ± 0.03
Lysine	1.79 ± 0.12	2.73 ± 0.13 ^a^	2.24 ± 0.15 ^d^	2.36 ± 0.19 ^c^
Methionine	0.17 ± 0.06	0.28 ± 0.06	0.18 ± 0.05	0.21 ± 0.05
Phenylalanine	0.37 ± 0.01	0.39 ± 0.02	0.36 ± 0.02	0.41 ± 0.02
Threonine	1.02 ± 0.06	1.62 ± 0.09 ^a^	1.20 ± 0.06 ^d^	1.09 ± 0.05 ^e^
Tryptophan	15.3 ± 0.5	16.0 ± 0.5	15.1 ± 0.8	15.4 ± 0.5
**Sum EAA**	**21.1 ± 0.5**	**24.5 ± 0.8 ^a^**	**21.8 ± 1.1 ^d^**	**22.5 ± 0.6**
Alanine	8.58 ± 2.14	7.84 ± 1.18	7.74 ± 1.28	7.90 ± 0.96
Arginine	0.83 ± 0.06	0.79 ± 0.04	0.85 ± 0.06	0.84 ± 0.04
Asparagine	0.36 ± 0.03	0.40 ± 0.01	0.38 ± 0.02	0.39 ± 0.03
Aspartic acid	0.87 ± 0.09	1.23 ± 0.09 ^a^	1.10 ± 0.09	1.26 ± 0.09 ^c^
Citrulline	0.45 ± 0.05	0.41 ± 0.04	0.38 ± 0.05	0.39 ± 0.04
Glutamine	5.26 ± 0.42	5.85 ± 0.29	5.54 ± 0.12	5.74 ± 0.30
Glutamic acid	4.44 ± 0.41	5.47 ± 0.25	4.83 ± 0.30	4.56 ± 0.23
Glycine	3.53 ± 0.11	3.41 ± 0.11	3.43 ± 0.12	3.43 ± 0.13
Serine	1.02 ± 0.07	1.02 ± 0.07	0.94 ± 0.04	0.99 ± 0.05
Tyrosine	0.38 ± 0.03	0.62 ± 0.03 ^a^	0.56 ± 0.04	0.73 ± 0.07 ^cf^
**Sum NEAA**	**25.7 ± 2.2**	**27.0 ± 1.4**	**25.8 ± 1.3**	**26.2 ± 0.7**
**Sum CEAA**	**11.0 ± 0.4**	**11.7 ± 0.4**	**11.3 ± 0.3**	**11.7 ± 0.5**
**Total AA**	**46.8 ± 2.62**	**51.5 ± 1.9**	**45.6 ± 2.3**	**48.7 ± 1.2**
